# Supplementary material for: Covalent linkage of the DNA repair template to the CRISPR-Cas9 nuclease enhances homology-directed repair
Source: eLife. 2018 May 29;7:e33761. doi: 10.7554/eLife.33761 (PMC6023611; doi:10.7554/eLife.33761)
Supplement: Supplementary file 2. — Allele plots show insertion/deletion variant alleles with frequency of at least 0.01%, and non-indel variants with frequency of at least 0.05% in any sample. When more than 50 variants passed these criteria, the top 50 alleles according to their maximum frequency in any sample are shown. From top to bottom, the consensus sequences for variant alleles are displayed in the order: no variant, precisely corrected allele, insertions (I) and deletions (D), single nucleotide variants (SNVs) and non-linear alignments. SNVs are only shown for non-indel variants and appear in color. In the y-axis labels, nucleotide numbers indicate the distance to the cut site. Variants are labelled with respect to the leftmost base. For example −5:9D is a 9 base pair deletion starting 5 bases upstream of the cut site. SNV labels show the bases that differ between the non-indel reads and the reference. The most common inserted sequences with less than 20 base pairs are shown in full in the legend. For longer and less frequent insertions the length is indicated. In the heatmap at right, the header shows the number of merged read pairs with alignments spanning the guide sequence. The x-axis is coloured according to experimental replicate. [file elife-33761-supp2.pdf]

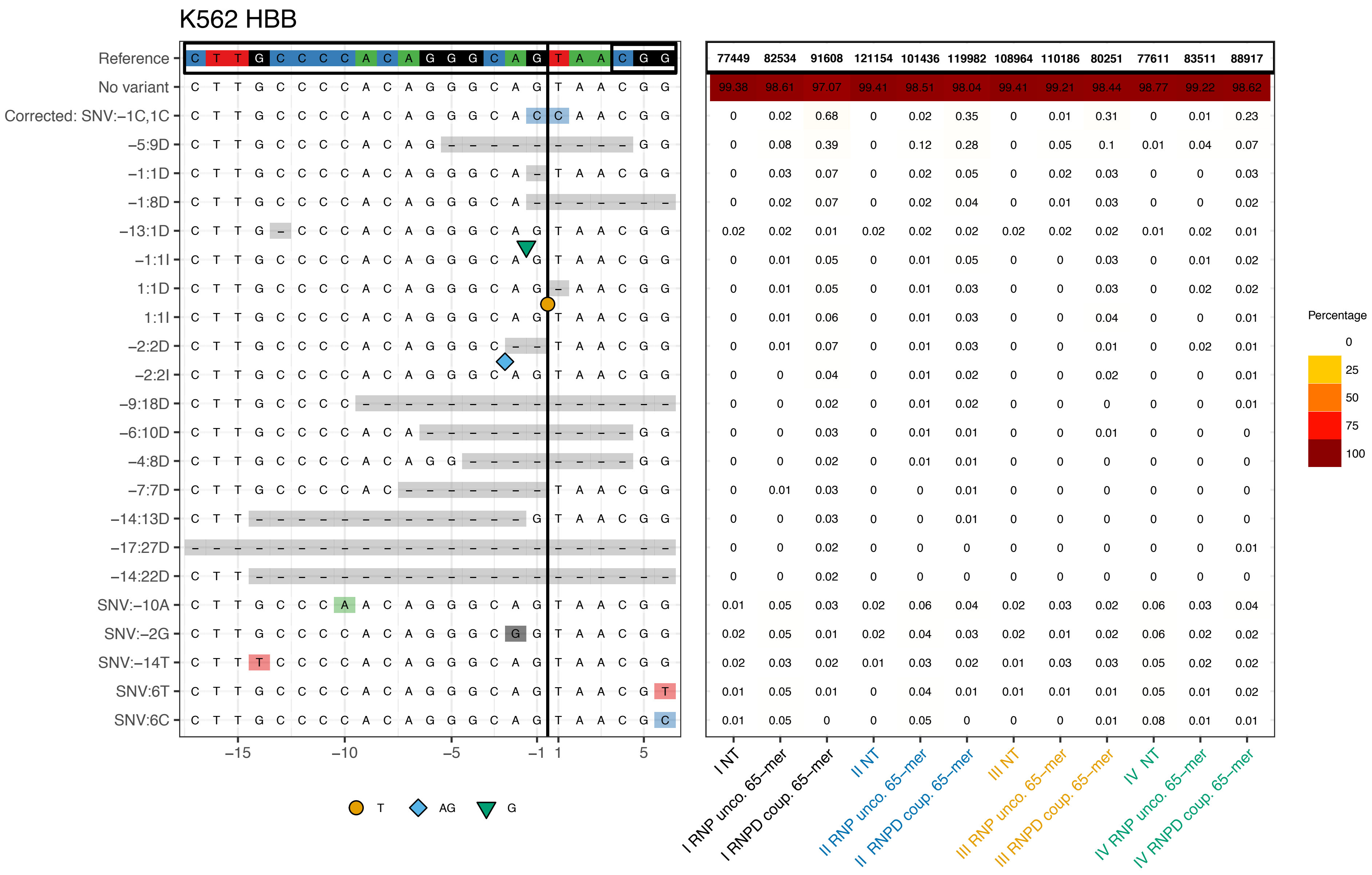
K562 HBB
Reference
No variant
Corrected: SNV:–1C,1C
–5:9D
–1:1D
–1:8D
–13:1D
–1:1I
1:1D
1:1I
–2:2D
–2:2I
–9:18D
–6:10D
–4:8D
–7:7D
–14:13D
–17:27D
–14:22D
SNV:–10A
SNV:–2G
SNV:–14T
SNV:6T
SNV:6C
–15
–10
–5
–1
1
5
T
AG
G
77449 82534 91608 121154 101436 119982 108964 110186 80251 77611 83511 88917
99.38 98.61 97.07 99.41 98.51 98.04 99.41 99.21 98.44 98.77 99.22 98.62
0 0.02 0.68 0 0.02 0.35 0 0.01 0.31 0 0.01 0.23
0 0.08 0.39 0 0.12 0.28 0 0.05 0.1 0.01 0.04 0.07
0 0.03 0.07 0 0.02 0.05 0 0.02 0.03 0 0 0.03
0 0.02 0.07 0 0.02 0.04 0 0.01 0.03 0 0 0.02
0.02 0.02 0.01 0.02 0.02 0.02 0.02 0.02 0.02 0.01 0.02 0.01
0 0.01 0.05 0 0.01 0.05 0 0 0.03 0 0.01 0.02
0 0.01 0.05 0 0.01 0.03 0 0 0.03 0 0.02 0.02
0 0.01 0.06 0 0.01 0.03 0 0 0.04 0 0 0.01
0 0.01 0.07 0 0.01 0.03 0 0 0.01 0 0.02 0.01
0 0 0.04 0 0.01 0.02 0 0 0.02 0 0 0.01
0 0 0.02 0 0.01 0.02 0 0 0 0 0 0.01
0 0 0.03 0 0.01 0.01 0 0 0.01 0 0 0
0 0 0.02 0 0.01 0.01 0 0 0 0 0 0
0 0.01 0.03 0 0 0.01 0 0 0 0 0 0
0 0 0.03 0 0 0.01 0 0 0 0 0 0
0 0 0.02 0 0 0 0 0 0 0 0 0.01
0 0 0.02 0 0 0 0 0 0 0 0 0
0.01 0.05 0.03 0.02 0.06 0.04 0.02 0.03 0.02 0.06 0.03 0.04
0.02 0.05 0.01 0.02 0.04 0.03 0.02 0.01 0.02 0.06 0.02 0.02
0.02 0.03 0.02 0.01 0.03 0.02 0.01 0.03 0.03 0.05 0.02 0.02
0.01 0.05 0.01 0 0.04 0.01 0.01 0.01 0.01 0.05 0.01 0.02
0.01 0.05 0 0 0.05 0 0 0 0.01 0.08 0.01 0.01
I NT
I RNP unco. 65–mer
I RNPD coup. 65–mer
II NT
II RNP unco. 65–mer
II RNPD coup. 65–mer
III NT
III RNP unco. 65–mer
III RNPD coup. 65–mer
IV NT
IV RNP unco. 65–mer
IV RNPD coup. 65–mer
Percentage
0
25
50
75
100

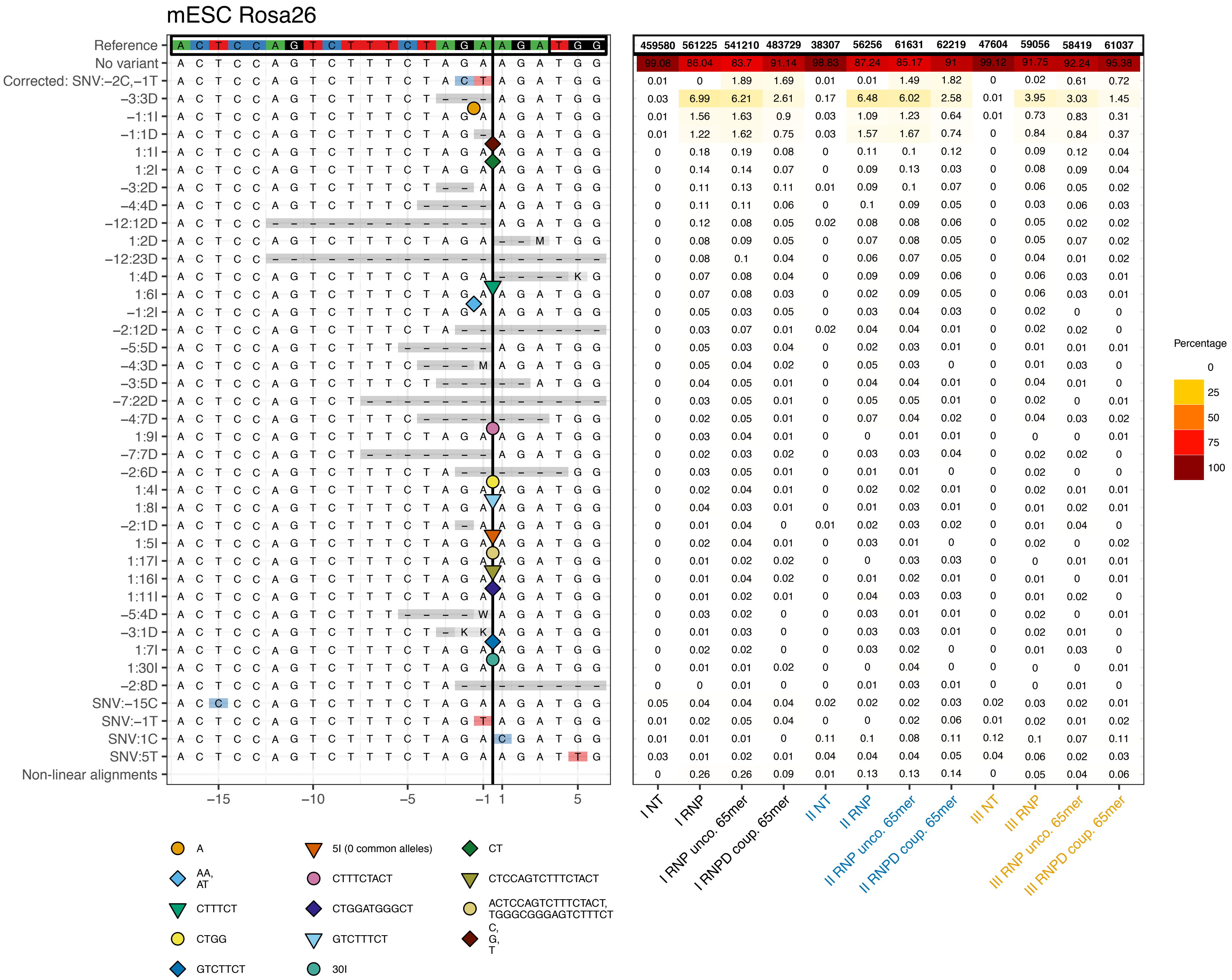

mESC Rosa26
Reference
No variant
Corrected: SNV:−2C,−1T
−3:3D
−1:1I
−1:1D
1:1I
1:2I
−3:2D
−4:4D
−12:12D
1:2D
−12:23D
1:4D
1:6I
−1:2I
−2:12D
−5:5D
−4:3D
−3:5D
−7:22D
−4:7D
1:9I
−7:7D
−2:6D
1:4I
1:8I
−2:1D
1:5I
1:17I
1:16I
1:11I
−5:4D
−3:1D
1:7I
1:30I
−2:8D
SNV:−15C
SNV:−1T
SNV:1C
SNV:5T
Non-linear alignments
459580 561225 541210 483729 38307 56256 61631 62219 47604 59056 58419 61037
I NT
I RNP
I RNP unco. 65mer
I RNPD coup. 65mer
II NT
II RNP
II RNP unco. 65mer
II RNPD coup. 65mer
III NT
III RNP
III RNP unco. 65mer
III RNPD coup. 65mer
Percentage
0
25
50
75
100
A
AA, AT
CTTTCT
CTGG
GTCTTCT
5I (0 common alleles)
CTTTCTACT
CTGGATGGGCT
GTCTTTCT
30I
CT
CTCCAGTCTTTCTACT
ACTCCAGTCTTTCTACT, TGGGCGGGAGTCTTTCT
C, G, T

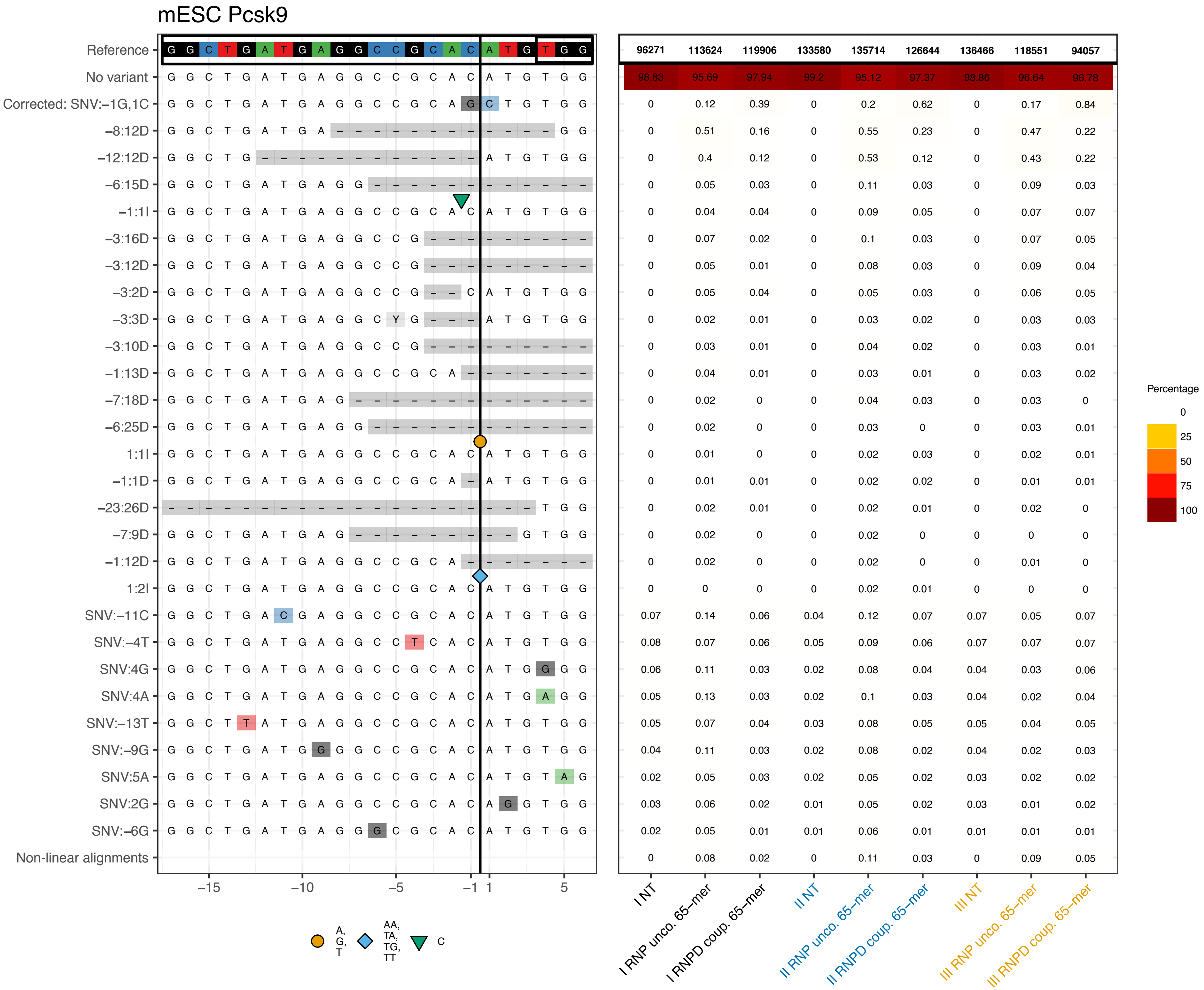

mESC Pcsk9
Reference
No variant
Corrected: SNV:−1G,1C
−8:12D
−12:12D
−6:15D
−1:1I
−3:16D
−3:12D
−3:2D
−3:3D
−3:10D
−1:13D
−7:18D
−6:25D
1:1I
−1:1D
−23:26D
−7:9D
−1:12D
1:2I
SNV:−11C
SNV:−4T
SNV:4G
SNV:4A
SNV:−13T
SNV:−9G
SNV:5A
SNV:2G
SNV:−6G
Non-linear alignments
−15
−10
−5
−1
1
5
96271
113624
119906
133580
135714
126644
136466
118551
94057
I NT
I RNP unco. 65−mer
I RNPD coup. 65−mer
II NT
II RNP unco. 65−mer
II RNPD coup. 65−mer
III NT
III RNP unco. 65−mer
III RNPD coup. 65−mer
Percentage
0
25
50
75
100
A, G, T
AA, TA, TG, TT
C

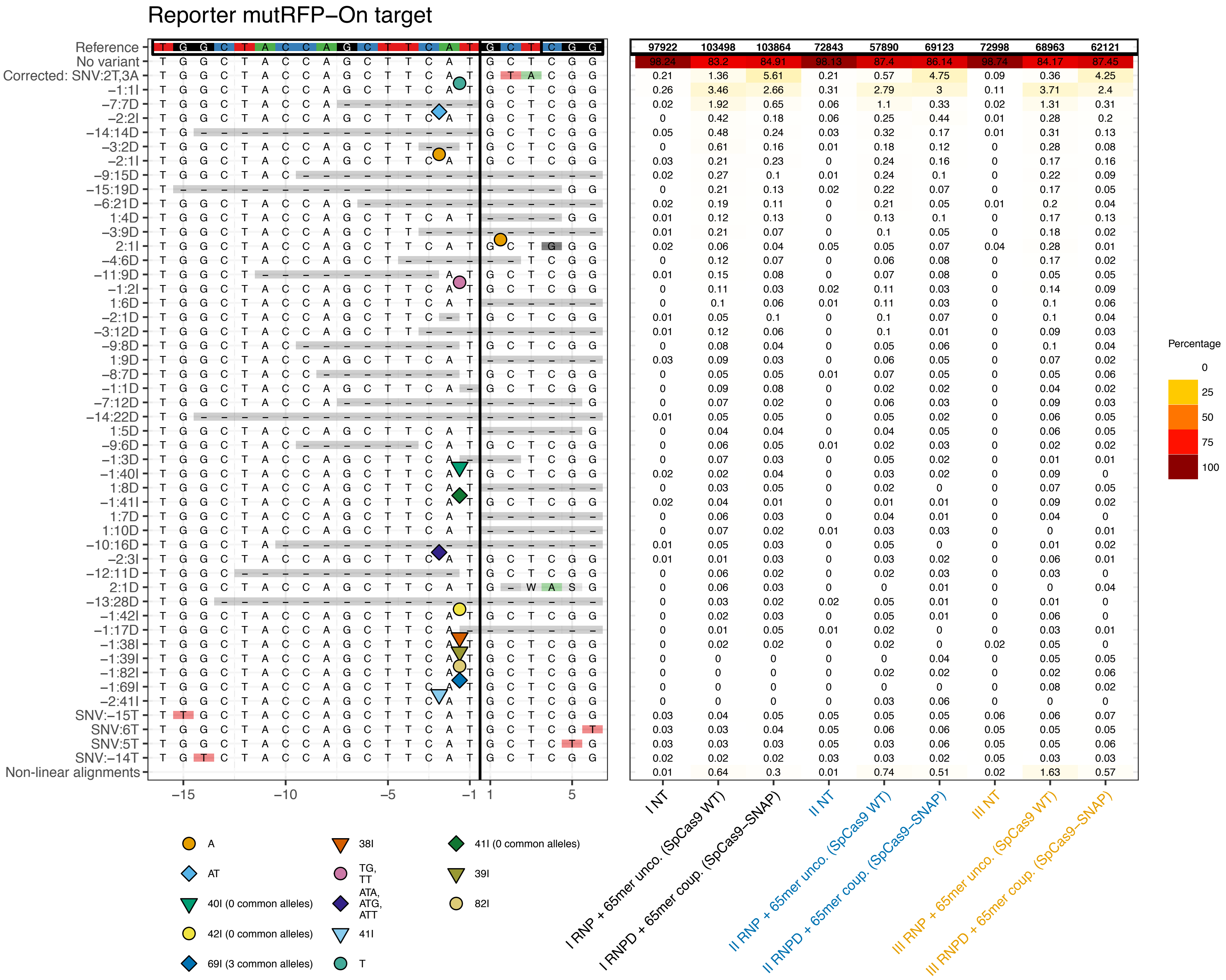

Reporter mutRFP−On target
Reference
No variant
Corrected: SNV:2T,3A
−1:1I
−7:7D
−2:2I
−14:14D
−3:2D
−2:1I
−9:15D
−15:19D
−6:21D
1:4D
−3:9D
2:1I
−4:6D
−11:9D
−1:2I
1:6D
−2:1D
−3:12D
−9:8D
1:9D
−8:7D
−1:1D
−7:12D
−14:22D
1:5D
−9:6D
−1:3D
−1:40I
1:8D
−1:41I
1:7D
1:10D
−10:16D
−2:3I
−12:11D
2:1D
−13:28D
−1:42I
−1:17D
−1:38I
−1:39I
−1:82I
−1:69I
−2:41I
SNV:−15T
SNV:6T
SNV:5T
SNV:−14T
Non-linear alignments
−15
−10
−5
−1
1
5
97922
103498
103864
72843
57890
69123
72998
68963
62121
I NT
I RNP + 65mer unco. (SpCas9 WT)
I RNPD + 65mer coup. (SpCas9–SNAP)
II NT
II RNP + 65mer unco. (SpCas9 WT)
II RNPD + 65mer coup. (SpCas9–SNAP)
III NT
III RNP + 65mer unco. (SpCas9 WT)
III RNPD + 65mer coup. (SpCas9–SNAP)
Percentage
0
25
50
75
100
A
AT
40I (0 common alleles)
42I (0 common alleles)
69I (3 common alleles)
38I
TG, TT
ATA, ATG, ATT
41I
T
41I (0 common alleles)
39I
82I

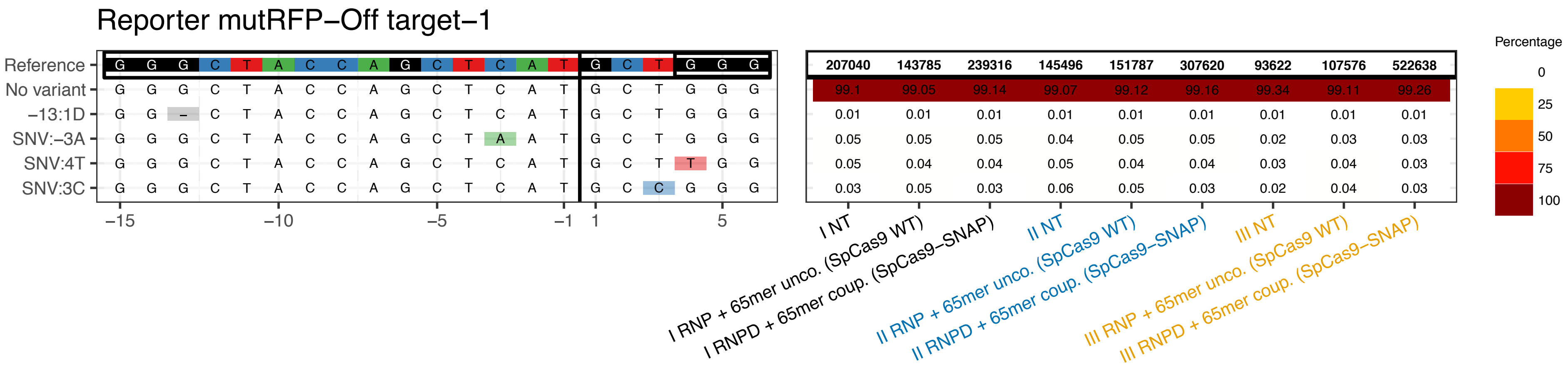

Reporter mutRFP–Off target–1
Reference
No variant
–13:1D
SNV:–3A
SNV:4T
SNV:3C
–15
–10
–5
–1
1
5
207040
143785
239316
145496
151787
307620
93622
107576
522638
99.1
99.05
99.14
99.07
99.12
99.16
99.34
99.11
99.26
I NT
I RNP + 65mer unco. (SpCas9 WT)
I RNPD + 65mer coup. (SpCas9–SNAP)
II NT
II RNP + 65mer unco. (SpCas9 WT)
II RNPD + 65mer coup. (SpCas9–SNAP)
III NT
III RNP + 65mer unco. (SpCas9 WT)
III RNPD + 65mer coup. (SpCas9–SNAP)
Percentage
0
25
50
75
100

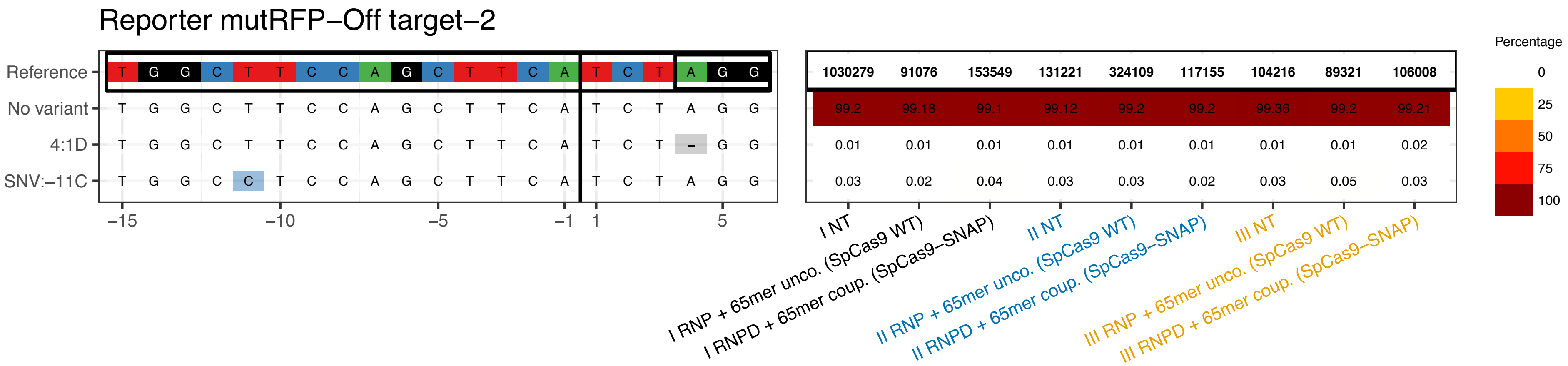

Reporter mutRFP−Off target−2
Reference
No variant
4:1D
SNV:−11C
T G G C T T C C A G C T T C A T C T A G G
−15
−10
−5
−1
1
5
1030279
91076
153549
131221
324109
117155
104216
89321
106008
99.2
99.18
99.1
99.12
99.2
99.2
99.36
99.2
99.21
0.01
0.01
0.01
0.01
0.01
0.01
0.01
0.01
0.02
0.03
0.02
0.04
0.03
0.03
0.02
0.03
0.05
0.03
I NT
I RNP + 65mer unco. (SpCas9 WT)
I RNPD + 65mer coup. (SpCas9−SNAP)
II NT
II RNP + 65mer unco. (SpCas9 WT)
II RNPD + 65mer coup. (SpCas9−SNAP)
III NT
III RNP + 65mer unco. (SpCas9 WT)
III RNPD + 65mer coup. (SpCas9−SNAP)
Percentage
0
25
50
75
100

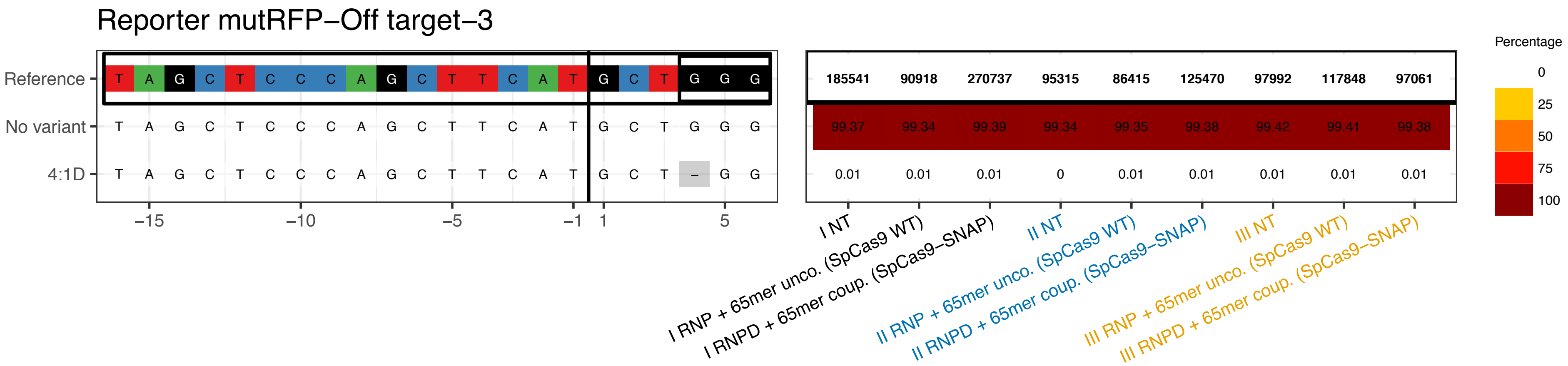
Reporter mutRFP−Off target−3
Reference
No variant
4:1D
T A G C T C C C A G C T T C A T G C T G G G
T A G C T C C C A G C T T C A T G C T G G G
T A G C T C C C A G C T T C A T G C T – G G
−15
−10
−5
−1
1
5
185541
90918
270737
95315
86415
125470
97992
117848
97061
99.37
99.34
99.39
99.34
99.35
99.38
99.42
99.41
99.38
0.01
0.01
0.01
0
0.01
0.01
0.01
0.01
0.01
I NT
I RNP + 65mer unco. (SpCas9 WT)
I RNPD + 65mer coup. (SpCas9−SNAP)
II NT
II RNP + 65mer unco. (SpCas9 WT)
II RNPD + 65mer coup. (SpCas9−SNAP)
III NT
III RNP + 65mer unco. (SpCas9 WT)
III RNPD + 65mer coup. (SpCas9−SNAP)
Percentage
0
25
50
75
100

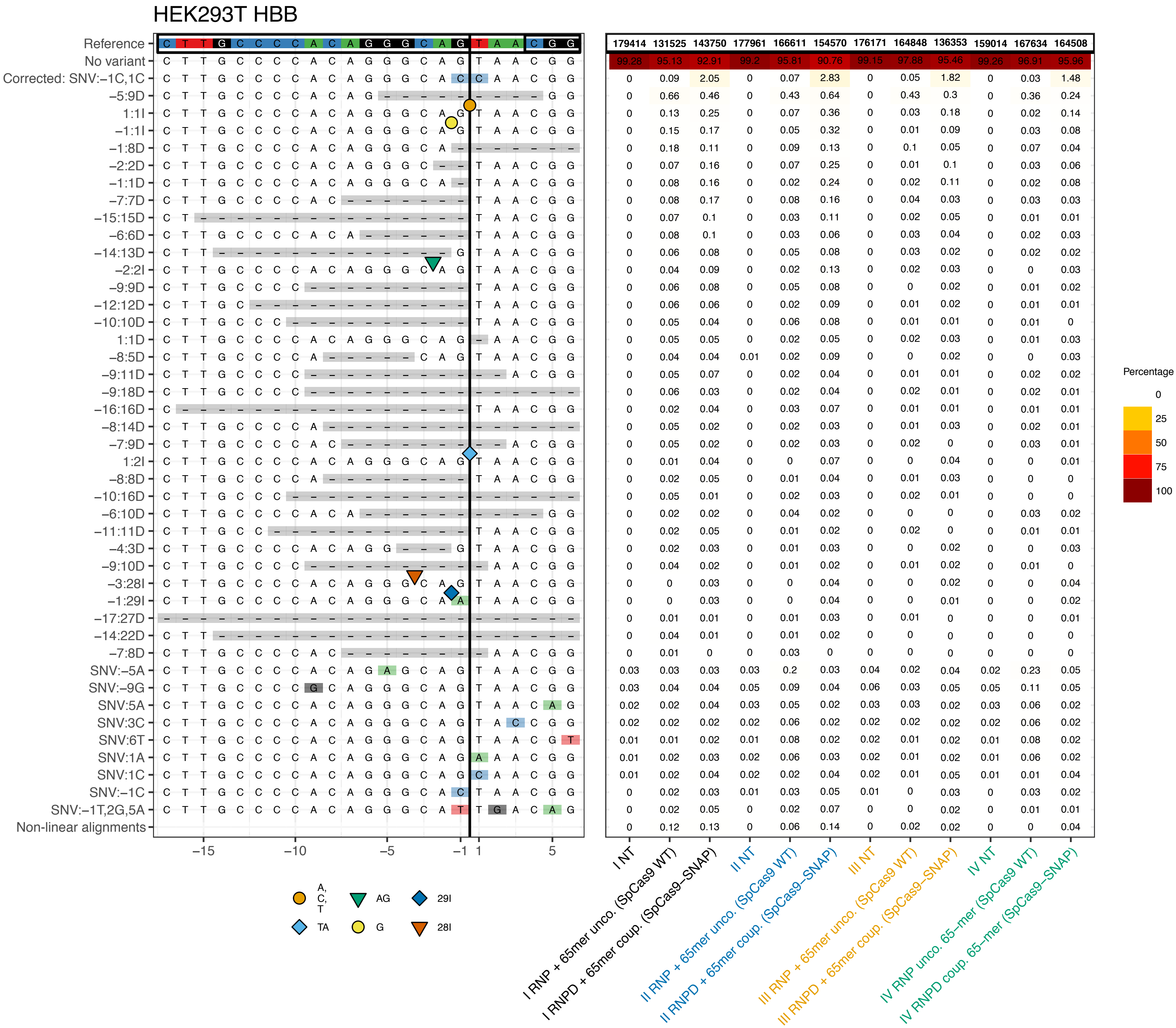
HEK293T HBB
Reference
No variant
Corrected: SNV:−1C,1C
−5:9D
1:1I
−1:1I
−1:8D
−2:2D
−1:1D
−7:7D
−15:15D
−6:6D
−14:13D
−2:2I
−9:9D
−12:12D
−10:10D
1:1D
−8:5D
−9:11D
−9:18D
−16:16D
−8:14D
−7:9D
1:2I
−8:8D
−10:16D
−6:10D
−11:11D
−4:3D
−9:10D
−3:28I
−1:29I
−17:27D
−14:22D
−7:8D
SNV:−5A
SNV:−9G
SNV:5A
SNV:3C
SNV:6T
SNV:1A
SNV:1C
SNV:−1C
SNV:−1T,2G,5A
Non-linear alignments
−15
−10
−5
−1
1
5
A, C, T
AG
29I
TA
G
28I
179414
131525
143750
177961
166611
154570
176171
164848
136353
159014
167634
164508
Percentage
0
25
50
75
100
I NT
I RNP + 65mer unco. (SpCas9 WT)
I RNPD + 65mer coup. (SpCas9–SNAP)
II NT
II RNP + 65mer unco. (SpCas9 WT)
II RNPD + 65mer coup. (SpCas9–SNAP)
III NT
III RNP + 65mer unco. (SpCas9 WT)
III RNPD + 65mer coup. (SpCas9–SNAP)
IV NT
IV RNP unco. 65–mer (SpCas9 WT)
IV RNPD coup. 65–mer (SpCas9–SNAP)

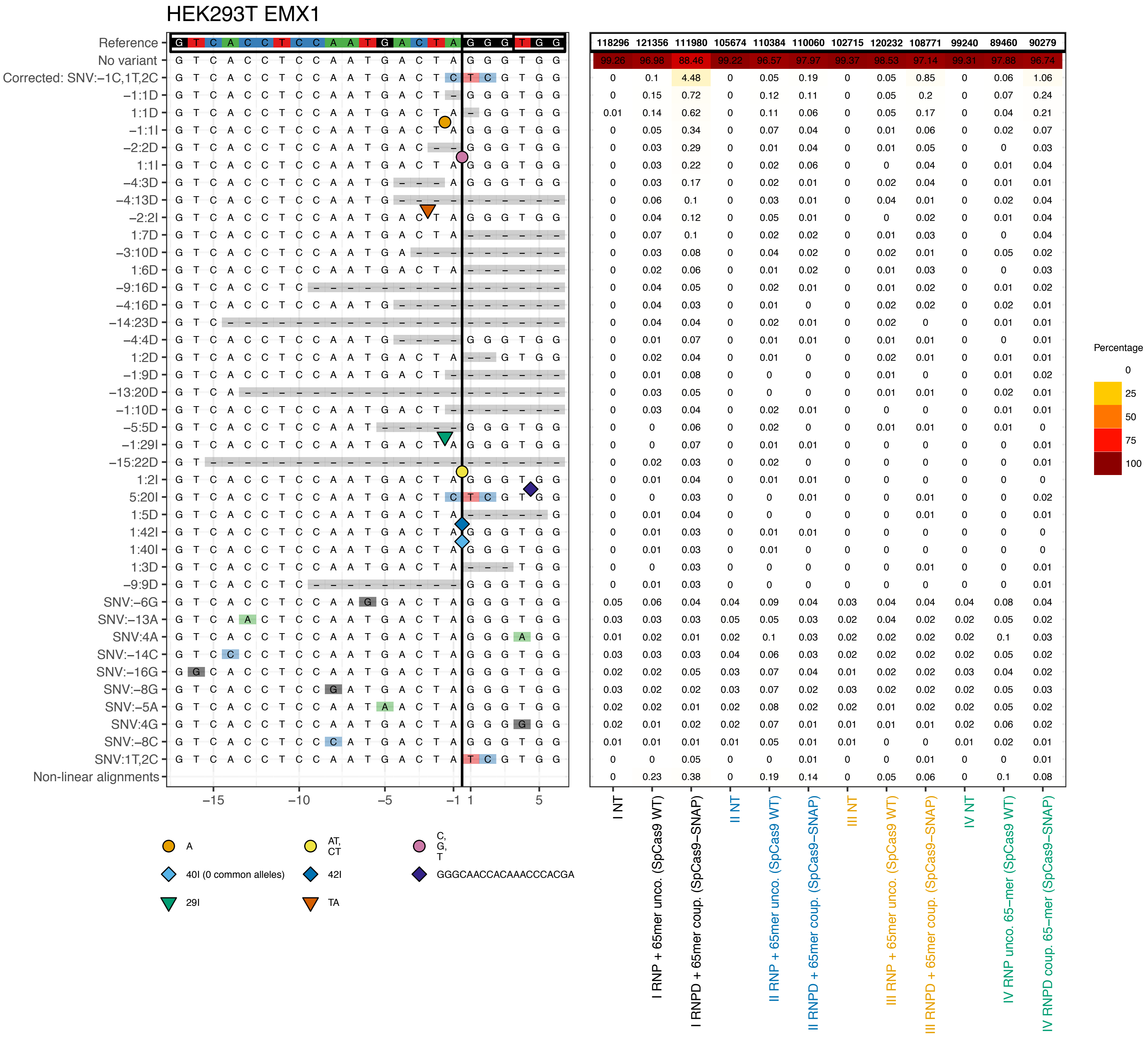
HEK293T EMX1
Reference
No variant
Corrected: SNV:−1C,1T,2C
−1:1D
1:1D
−1:1I
−2:2D
1:1I
−4:3D
−4:13D
−2:2I
1:7D
−3:10D
1:6D
−9:16D
−4:16D
−14:23D
−4:4D
1:2D
−1:9D
−13:20D
−1:10D
−5:5D
−1:29I
−15:22D
1:2I
5:20I
1:5D
1:42I
1:40I
1:3D
−9:9D
SNV:−6G
SNV:−13A
SNV:4A
SNV:−14C
SNV:−16G
SNV:−8G
SNV:−5A
SNV:4G
SNV:−8C
SNV:1T,2C
Non-linear alignments
G T C A C C T C C A A T G A C T A G G G T G G
−15 −10 −5 −1 1 5
118296 121356 111980 105674 110384 110060 102715 120232 108771 99240 89460 90279
99.26 96.98 88.46 99.22 96.57 97.97 99.37 98.53 97.14 99.31 97.88 96.74
0 0.1 4.48 0 0.05 0.19 0 0.05 0.85 0 0.06 1.06
0 0.15 0.72 0 0.12 0.11 0 0.05 0.2 0 0.07 0.24
0.01 0.14 0.62 0 0.11 0.06 0 0.05 0.17 0 0.04 0.21
0 0.05 0.34 0 0.07 0.04 0 0.01 0.06 0 0.02 0.07
0 0.03 0.29 0 0.01 0.04 0 0.01 0.05 0 0 0.03
0 0.03 0.22 0 0.02 0.06 0 0 0.04 0 0.01 0.04
0 0.03 0.17 0 0.02 0.01 0 0.02 0.04 0 0.01 0.01
0 0.06 0.1 0 0.03 0.01 0 0.04 0.01 0 0.02 0.04
0 0.04 0.12 0 0.05 0.01 0 0 0.02 0 0.01 0.04
0 0.07 0.1 0 0.02 0.02 0 0.01 0.03 0 0 0.04
0 0.03 0.08 0 0.04 0.02 0 0.02 0.01 0 0.05 0.02
0 0.02 0.06 0 0.01 0.02 0 0.01 0.03 0 0 0.03
0 0.04 0.05 0 0.02 0.01 0 0.01 0.02 0 0.01 0.02
0 0.04 0.03 0 0.01 0 0 0.02 0.02 0 0.02 0.01
0 0.04 0.04 0 0.02 0.01 0 0.02 0 0 0.01 0.01
0 0.01 0.07 0 0.01 0.01 0 0.01 0.01 0 0 0.01
0 0.02 0.04 0 0.01 0 0 0.02 0.01 0 0.01 0.01
0 0.01 0.08 0 0 0 0 0.01 0 0 0.01 0.02
0 0.03 0.05 0 0 0 0 0.01 0.01 0 0.02 0.01
0 0.03 0.04 0 0.02 0.01 0 0 0 0 0.01 0.01
0 0 0.06 0 0.02 0 0 0.01 0.01 0 0.01 0
0 0 0.07 0 0.01 0.01 0 0 0 0 0 0.01
0 0.02 0.03 0 0.02 0 0 0 0 0 0 0.01
0 0.01 0.04 0 0.01 0.01 0 0 0 0 0 0
0 0 0.03 0 0 0.01 0 0 0.01 0 0 0.02
0 0.01 0.04 0 0 0 0 0 0.01 0 0 0.01
0 0.01 0.03 0 0.01 0.01 0 0 0 0 0 0
0 0.01 0.03 0 0.01 0 0 0 0 0 0 0
0 0 0.03 0 0 0 0 0 0.01 0 0 0.01
0 0.01 0.03 0 0 0 0 0 0 0 0 0.01
0.05 0.06 0.04 0.04 0.09 0.04 0.03 0.04 0.04 0.04 0.08 0.04
0.03 0.03 0.03 0.05 0.05 0.03 0.02 0.04 0.02 0.02 0.05 0.02
0.01 0.02 0.01 0.02 0.1 0.03 0.02 0.02 0.02 0.02 0.1 0.03
0.03 0.03 0.03 0.04 0.06 0.03 0.02 0.02 0.02 0.02 0.05 0.02
0.02 0.02 0.05 0.03 0.07 0.04 0.01 0.02 0.02 0.03 0.04 0.02
0.03 0.02 0.02 0.03 0.07 0.02 0.03 0.02 0.02 0.02 0.05 0.03
0.02 0.02 0.01 0.02 0.08 0.02 0.02 0.01 0.02 0.02 0.05 0.02
0.02 0.01 0.02 0.02 0.07 0.01 0.01 0.01 0.01 0.02 0.06 0.02
0.01 0.01 0.01 0.01 0.05 0.01 0.01 0 0 0.01 0.02 0.01
0 0 0.05 0 0 0.01 0 0 0.01 0 0 0.01
0 0.23 0.38 0 0.19 0.14 0 0.05 0.06 0 0.1 0.08
I NT
I RNP + 65mer unco. (SpCas9 WT)
I RNPD + 65mer coup. (SpCas9–SNAP)
II NT
II RNP + 65mer unco. (SpCas9 WT)
II RNPD + 65mer coup. (SpCas9–SNAP)
III NT
III RNP + 65mer unco. (SpCas9 WT)
III RNPD + 65mer coup. (SpCas9–SNAP)
IV NT
IV RNP unco. 65–mer (SpCas9 WT)
IV RNPD coup. 65–mer (SpCas9–SNAP)
Percentage
0
25
50
75
100
A
AT, CT
C, G, T
40I (0 common alleles)
42I
GGGCAACCACAAACCCACGA
29I
TA

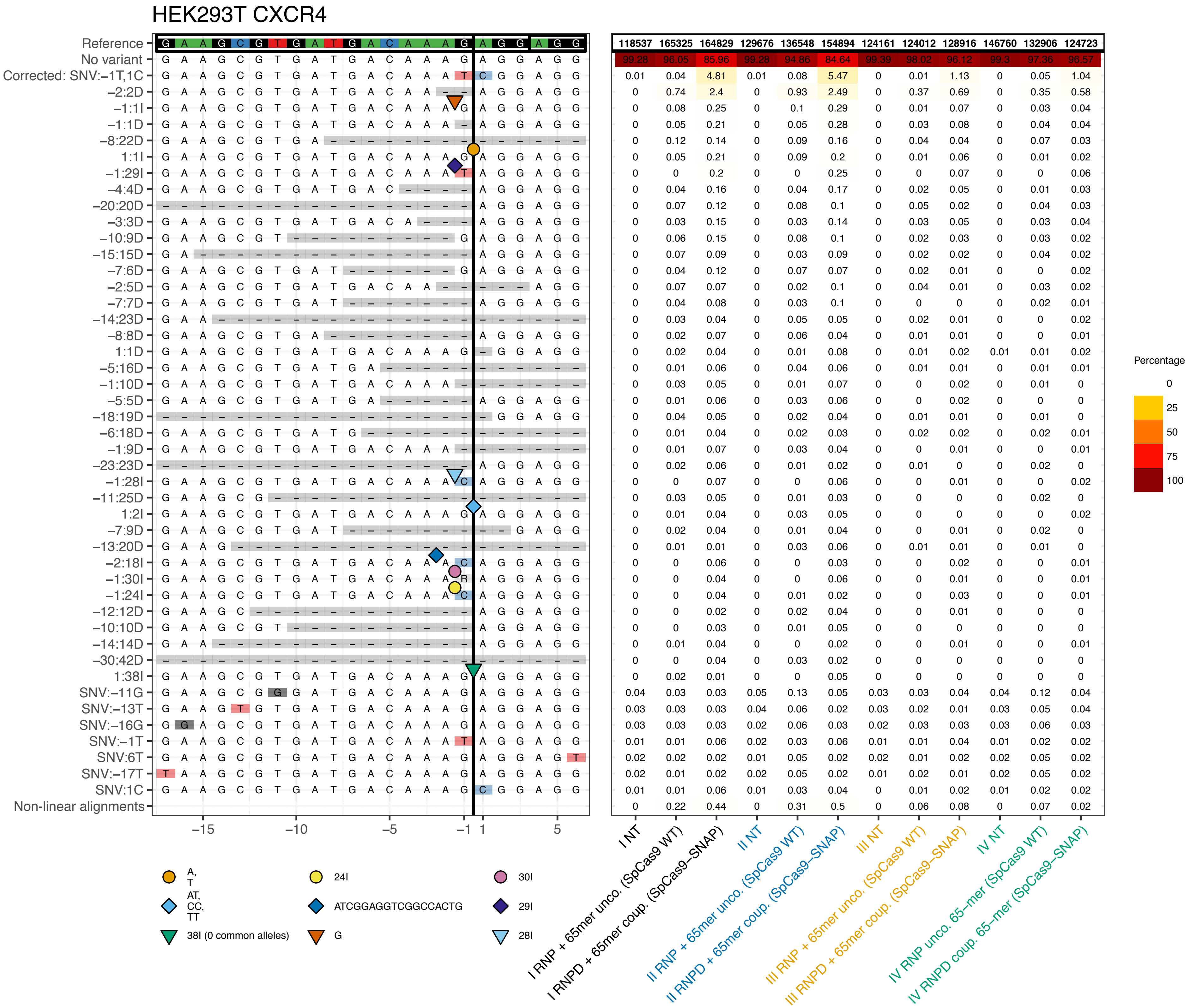
HEK293T CXCR4
Reference
118537 165325 164829 129676 136548 154894 124161 124012 128916 146760 132906 124723
No variant 99.28 96.05 85.96 99.28 94.86 84.64 99.39 98.02 96.12 99.3 97.36 96.57
Corrected: SNV:−1T,1C 0.01 0.04 4.81 0.01 0.08 5.47 0 0.01 1.13 0 0.05 1.04
−2:2D 0 0.74 2.4 0 0.93 2.49 0 0.37 0.69 0 0.35 0.58
−1:1I 0 0.08 0.25 0 0.1 0.29 0 0.01 0.07 0 0.03 0.04
−1:1D 0 0.05 0.21 0 0.05 0.28 0 0.03 0.08 0 0.04 0.04
−8:22D 0 0.12 0.14 0 0.09 0.16 0 0.04 0.04 0 0.07 0.03
1:1I 0 0.05 0.21 0 0.09 0.2 0 0.01 0.06 0 0.01 0.02
−1:29I 0 0 0.2 0 0 0.25 0 0 0.07 0 0 0.06
−4:4D 0 0.04 0.16 0 0.04 0.17 0 0.02 0.05 0 0.01 0.03
−20:20D 0 0.07 0.12 0 0.08 0.1 0 0.05 0.02 0 0.04 0.03
−3:3D 0 0.03 0.15 0 0.03 0.14 0 0.03 0.05 0 0.03 0.04
−10:9D 0 0.06 0.15 0 0.08 0.1 0 0.02 0.03 0 0.03 0.02
−15:15D 0 0.07 0.09 0 0.03 0.09 0 0.02 0.02 0 0.04 0.02
−7:6D 0 0.04 0.12 0 0.07 0.07 0 0.02 0.01 0 0 0.02
−2:5D 0 0.07 0.07 0 0.02 0.1 0 0.04 0.01 0 0.03 0.02
−7:7D 0 0.04 0.08 0 0.03 0.1 0 0 0 0 0.02 0.01
−14:23D 0 0.03 0.04 0 0.05 0.05 0 0.02 0.01 0 0 0.02
−8:8D 0 0.02 0.07 0 0.06 0.04 0 0.01 0.01 0 0 0.01
1:1D 0 0.02 0.04 0 0.01 0.08 0 0.01 0.02 0.01 0.01 0.02
−5:16D 0 0.01 0.06 0 0.04 0.06 0 0.01 0.01 0 0.01 0.01
−1:10D 0 0.03 0.05 0 0.01 0.07 0 0 0.02 0 0.01 0
−5:5D 0 0.01 0.06 0 0.03 0.06 0 0 0.02 0 0 0
−18:19D 0 0.04 0.05 0 0.02 0.04 0 0.01 0.01 0 0.01 0
−6:18D 0 0.01 0.04 0 0.02 0.03 0 0.02 0.02 0 0.02 0.01
−1:9D 0 0.01 0.07 0 0.03 0.04 0 0 0.01 0 0 0.01
−23:23D 0 0.02 0.06 0 0.01 0.02 0 0.01 0 0 0.02 0
−1:28I 0 0 0.07 0 0 0.06 0 0 0.01 0 0 0.02
−11:25D 0 0.03 0.05 0 0.01 0.03 0 0 0 0 0.02 0
1:2I 0 0.01 0.04 0 0.03 0.05 0 0 0 0 0 0.02
−7:9D 0 0.02 0.04 0 0.01 0.04 0 0 0.01 0 0.02 0
−13:20D 0 0.01 0.01 0 0.03 0.06 0 0.01 0.01 0 0.01 0
−2:18I 0 0 0.06 0 0 0.03 0 0 0.02 0 0 0.01
−1:30I 0 0 0.04 0 0 0.06 0 0 0.01 0 0 0.01
−1:24I 0 0 0.04 0 0.01 0.02 0 0 0.03 0 0 0.01
−12:12D 0 0 0.02 0 0.02 0.04 0 0 0.01 0 0 0
−10:10D 0 0 0.03 0 0.01 0.05 0 0 0 0 0 0
−14:14D 0 0.01 0.04 0 0 0.02 0 0 0.01 0 0 0.01
−30:42D 0 0 0.04 0 0.03 0.02 0 0 0 0 0 0
1:38I 0 0.02 0.01 0 0 0.05 0 0 0 0 0 0
SNV:−11G 0.04 0.03 0.03 0.05 0.13 0.05 0.03 0.03 0.04 0.04 0.12 0.04
SNV:−13T 0.03 0.03 0.03 0.04 0.06 0.02 0.03 0.02 0.01 0.03 0.05 0.04
SNV:−16G 0.03 0.03 0.03 0.02 0.06 0.03 0.02 0.03 0.03 0.03 0.06 0.03
SNV:−1T 0.01 0.01 0.06 0.02 0.03 0.06 0.01 0.01 0.04 0.01 0.02 0.02
SNV:6T 0.02 0.02 0.02 0.01 0.05 0.02 0.02 0.01 0.02 0.02 0.05 0.02
SNV:−17T 0.02 0.01 0.02 0.02 0.05 0.02 0.01 0.02 0.01 0.02 0.05 0.02
SNV:1C 0.01 0.01 0.06 0.01 0.03 0.04 0 0.01 0.02 0.01 0.02 0.02
Non-linear alignments 0 0.22 0.44 0 0.31 0.5 0 0.06 0.08 0 0.07 0.02
−15 −10 −5 −1 1 5
I NT
I RNP + 65mer unco. (SpCas9 WT)
I RNPD + 65mer coup. (SpCas9–SNAP)
II NT
II RNP + 65mer unco. (SpCas9 WT)
II RNPD + 65mer coup. (SpCas9–SNAP)
III NT
III RNP + 65mer unco. (SpCas9 WT)
III RNPD + 65mer coup. (SpCas9–SNAP)
IV NT
IV RNP unco. 65–mer (SpCas9 WT)
IV RNPD coup. 65–mer (SpCas9–SNAP)
Percentage
0
25
50
75
100
A, T
24I
30I
AT, CC, TT
ATCGGAGGTCGGCCACTG
29I
38I (0 common alleles)
G
28I
