# Supplementary material for: Covalent linkage of the DNA repair template to the CRISPR-Cas9 nuclease enhances homology-directed repair
Source: eLife. 2018 May 29;7:e33761. doi: 10.7554/eLife.33761 (PMC6023611; doi:10.7554/eLife.33761)
Supplement: Transparent reporting form [file elife-33761-transrepform.docx]

***eLife’s* transparent reporting form**

We encourage authors to provide detailed information *within their submission* to facilitate the interpretation and replication of experiments. If you have any questions, please contact us: [editorial@elifesciences.org](mailto:editorial@elifesciences.org).

**Sample-size estimation**

- You should state whether an appropriate sample size was computed when the study was being designed
- You should state the statistical method of sample size computation and any required assumptions
- If no explicit power analysis was used, you should describe how you decided what sample (replicate) size (number) to use

Please outline where this information can be found within the submission (e.g., page numbers or figure legends), or explain why this information doesn’t apply to your submission:

We did not perform a power analysis to compute the appropriate sample size. However, to raise the stringency of our results that support the central findings of our paper we performed all experiments in at least 3 independent biological replicates.

**Replicates**

- You should report how often each experiment was performed
- You should include a definition of biological versus technical replication
- The data obtained should be provided and sufficient information should be provided to indicate the number of independent biological and/or technical replicates
- If you encountered any outliers, you should describe how these were handled
- Criteria for exclusion/inclusion of data should be clearly stated
- High-throughput sequence data should be uploaded before submission, with a private link for reviewers provided (these are available from both GEO and ArrayExpress)

Please outline where this information can be found within the submission (e.g., page numbers or figure legends), or explain why this information doesn’t apply to your submission:

Details about the number of replicates can be found in the figure legends. All replicates are biological repeats. Thus experiments were performed independently on separate days. No outliers were removed. Scripts for mapping sequencing data, counting mutations and generating plots are available at <https://github.com/HLindsay/Savic_CRISPR_HDR>. Fastq files have been uploaded to ArrayExpress^36^, the accession number is E-MTAB-6808.

**Statistical reporting**

- Statistical analysis methods should be described and justified
- Raw data should be presented in figures whenever informative to do so (typically when N per group is less than 10)
- For each experiment, you should identify the statistical tests used, exact values of N, definitions of center, methods of multiple test correction, and dispersion and precision measures (e.g., mean, median, SD, SEM, confidence intervals; and, for the major substantive results, a measure of effect size (e.g., Pearson's r, Cohen's d)
- Report exact p-values wherever possible alongside the summary statistics and 95% confidence intervals. These should be reported for all key questions and not only when the p-value is less than 0.05.

All statistical tests performed have been T-tests. The number of replicates for each experiment is detailed in the Figure Legends. Exact p values have been stated in the Figure 3 Figure Source data 1, Figure 4 Source data 1, Figure 4 Source data 3, Figure 5 Source data 1 and Figure 6 Source data 1. The error bars on all bar charts show the SEM.

Please outline where this information can be found within the submission (e.g., page numbers or figure legends), or explain why this information doesn’t apply to your submission:

(For large datasets, or papers with a very large number of statistical tests, you may upload a single table file with tests, Ns, etc., with reference to page numbers in the manuscript.)

**Additional data files (“source data”)**

- We encourage you to upload relevant additional data files, such as numerical data that are represented as a graph in a figure, or as a summary table
- Where provided, these should be in the most useful format, and they can be uploaded as “Source data” files linked to a main figure or table
- Include model definition files including the full list of parameters used
- Include code used for data analysis (e.g., R, MatLab)
- Avoid stating that data files are “available upon request”

Please indicate the figures or tables for which source data files have been provided:

A Source Data (excel files: Figure 3 Figure Source data 1, Figure 4 Source data 1, Figure 4 Source data 2, Figure 4 Source data 3, Figure 5 Source data 1 and Figure 6 Source data 1) have been uploaded with the submission containing numerical data and exact p-values of all graphs shown in the figures and figure supplements. This Source Data files have been referenced where appropriate in the Figure legends. All the Supplementary Files 1-4 have been referenced in the manuscript accordingly. Fastq files have been uploaded to ArrayExpress^36^, the accession number is E-MTAB-6808.
